# The medial habenula: still neglected

**DOI:** 10.3389/fnhum.2013.00931

**Published:** 2014-01-17

**Authors:** Humsini Viswanath, Asasia Q. Carter, Philip R. Baldwin, David L. Molfese, Ramiro Salas

**Affiliations:** Menninger Department of Psychiatry and Behavioral Sciences, Baylor College of MedicineHouston, TX, USA

**Keywords:** medial habenula (MHb), tobacco, stria medullaris, nicotinic receptors, addiction

## Abstract

The habenula is a small, bilateral brain structure located at the dorsal end of the diencephalon. This structure sends projections to the dopaminergic striatum and receives inputs from the limbic forebrain, making the habenula a unique modulator of cross-talk between these brain regions. Despite strong interest in the habenula during the seventies and eighties (Herkenham and Nauta, 1977; Beckstead, 1979; Beckstead et al., 1979; Herkenham and Nauta, 1979; Caldecott-Hazard et al., 1988), interest waned due to lack of a clearly identifiable functional role. Following Matsumoto and Hikosaka's seminal work on the lateral habenula as a predictor of negative reward in monkeys, the habenula has undergone a resurgence of scientific interest. Matsumoto and Hikosaka demonstrated an increase in habenular neuron firing when monkeys did not receive an expected juice reward (Matsumoto and Hikosaka, 2007). Studies have shown that increased habenular activity inactivates dopaminergic cells in the Rostromedial Tegmental Nucleus (RMTg) through GABAergic mechanisms (Jhou et al., 2009a,b). Additional studies link habenular activity to the regulation of serotonin and norepinephrine, suggesting the habenula modulates multiple brain systems (Strecker and Rosengren, 1989; Amat et al., 2001). These discoveries ushered in a series of new studies that have refocused attention on the lateral habenula and the importance of this small brain structure (Bianco and Wilson, 2009; Jhou et al., 2009a; Matsumoto and Hikosaka, 2009; Sartorius et al., 2010; Savitz et al., 2011). Recently, Geisler and Trimble reviewed this renewed interest in: *The Lateral Habenula: No Longer Neglected* (Geisler and Trimble, 2008). While the lateral habenula (LHb) has been extensively studied, the anatomically and histochemically distinct medial habenula (MHb) remains largely understudied. This short review argues that the MHb is functionally important and should be studied more aggressively.

## The medial habenula

**Figure 1:**
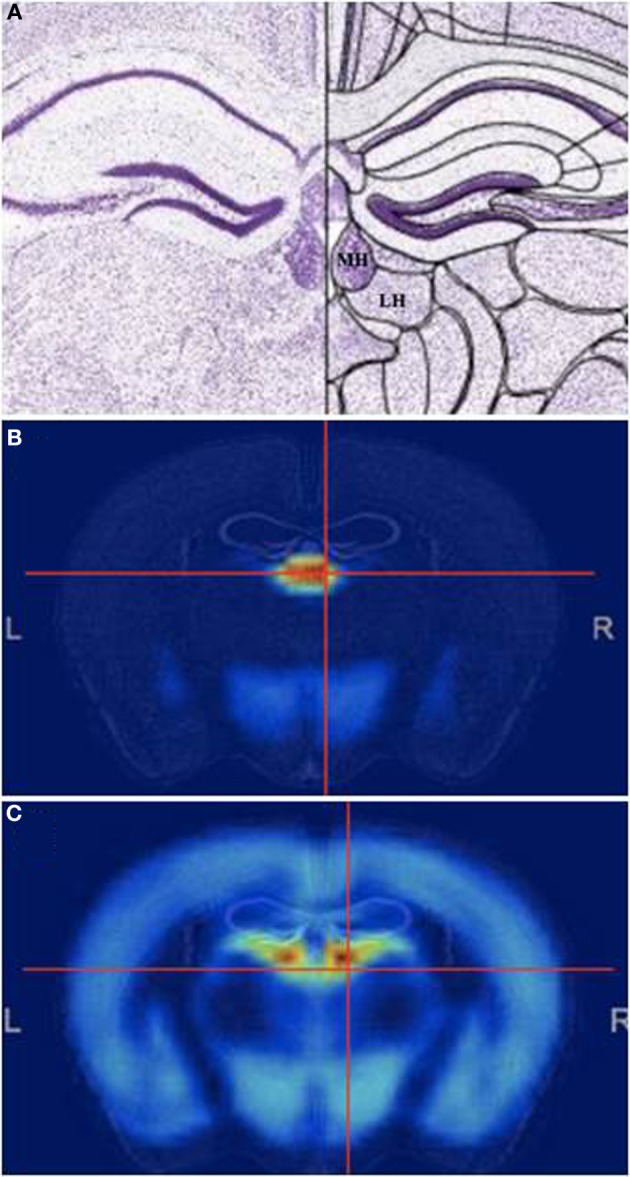
**The Medial habenula. (A)** Mouse coronal brain section at the level of the medial habenula, stained with Nissl. Medial (MHb) and lateral (LHb) are indicated. **(B,C)** Pattern of general genetic co-expression in the mouse brain using the MHb **(B)** or the LHb **(C)** as seeds. Note that the expression patterns are completely different. All figures from the Allen Brain Atlas (Lein et al., 2007).

The habenula (Latin for “little rein,” based on its shape) is a small, complex, and evolutionarily conserved structure. The habenular complex on the dorsal diencephalon is surrounded by the third ventricle and includes the medial habenula (MHb) and lateral habenula (LHb) (Figure 1). Both structures contain sub-nuclei, although structure-function relationships within these subdivisions have not been well characterized beyond localized expression of different neurotransmitter genes (Andres et al., 1999; Geisler et al., 2003). Gene expression in the MHb and LHb is very different, suggesting different functions (Figure 1). While the resurgence in habenula research in the last few years has focused primarily on the LHb, studies show that the MHb plays an important role in stress (Lecourtier et al., 2004; Mathuru and Jesuthasan, 2013), depression (Shumake et al., 2003), memory (Kobayashi et al., 2013), and nicotine withdrawal (Salas et al., 2009; Fowler et al., 2011; Frahm et al., 2011).

## Afferents and receptors

The MHb primarily receives inputs via the stria medullaris, a white matter pathway originating in the limbic system (Herkenham and Nauta, 1977; Qin and Luo, 2009). The most prominent MHb inputs are the triangular septal nucleus (TSN) and septofimbral nucleus (Herkenham and Nauta, 1977). Most posterior septum afferents terminate as cholinergic or substance P neurons, although inputs from the TSN may release glutamate (Glu) onto the habenula along with its ATP co-transmitter (Contestabile and Fonnum, 1983; Contestabile et al., 1987; Sperlagh et al., 1998; Lecourtier and Kelly, 2007; Qin and Luo, 2009)Many monoaminergic MHb inputs have been reported, including from the stem of the Ventral Tegmental Area (VTA), the mesencephalic raphe in the midbrain (Herkenham and Nauta, 1977), dopaminergic inputs from the interfascicular nucleus of VTA (Phillipson and Pycock, 1982), and noradrenergic inputs from the locus coeruleus and superior cervical ganglion (Gottesfeld, 1983). The MHb also receives GABAergic input from the Nucleus of Diagonal band in the basal forebrain and the medial septum (Qin and Luo, 2009). Interestingly, the MHb contains one of the highest concentrations of GABA(B) receptors in the brain, suggesting the presence of strong inhibitory inputs (Bischoff et al., 1999; Durkin et al., 1999; Charles et al., 2001; Wang et al., 2006). The MHb is also widely known for its abundance of nicotinic acetylcholine receptors (nAChRs). It is estimated that 90-100% of the neurons in the MHb express α3, α4, α5, β 2, and/or β 4 nA ChR subunits (Sheffield et al., 2000).

## Efferents and neurotransmitters

The MHb projects to the Interpeduncular Nucleus (IPN) via the internal portion of the fasciculus retroflexus (FR) (Herkenham and Nauta, 1979; Carlson et al., 2001) while the external portion of the FR axon bundle connects the LHb to the RMTg (Ellison, 2002). It has also been suggested that the MHb projects to the pineal gland and other midbrain structures (Rønnekleiv and Møller, 1979). The MHb projections to the IPN are particularly noteworthy because they contain three major output neurotransmitters: acetylcholine (Ach), Substance P, and Glu. Unlike the LHb, which diffusely expresses neurotransmitters across sub-nuclei, neurotransmitter expression in the MHb is highly localized. The superior MHb is glutamatergic and also expresses Interleukin-18 (IL-18); the dorsal-central MHb is both glutamatergic and substance P-ergic; and the inferior, ventral-center and lateral MHb are both cholinergic and glutamatergic (Aizawa et al., 2012). The restricted expression of these neurotransmitters to habenular sub-fields suggests a greater degree of functional specialization in the MHb than in the LHb. This specialization likely extends to projections from specific MHb sub-nuclei to downstream targets. It is likely that Glu projections from the MHb terminate in the IPN based on the high levels of cholinergic pre-synaptic markers and cholinergic receptors in this brain area (Hokfelt et al., 1975; Claudio Cuello et al., 1978; Brown et al., 1983; McGehee et al., 1995; Qin and Luo, 2009; Ren et al., 2011). The ACh and Substance P containing FR fibers project from the MHb through the IPN and then terminate in the VTA, possibly through an indirect pathway (Claudio Cuello et al., 1978; Balcita-Pedicino et al., 2011). Additional MHb projections continue beyond the IPN to the raphe nuclei in the brainstem. In turn, the raphe nuclei regulate serotonin levels throughout the brain (Groenewegen et al., 1986). Lastly, there is evidence of *boutons en passant* from the MHb to the LHb, but not from the LHb to the MHb (Kim and Chang, 2005). This directional connection suggests that the MHb may regulate LHb activity, while the LHb probably does not directly influence activity in the MHb. This needs to be further explored because the possible functional implications are important: it is possible that the MHb is one more input for the LHb, thus drugs and events that alter MHb activity would indirectly impinge on dopamine levels in the striatum. Although the function of the MHb is not likely to only provide input to the LHb, it is possible that at least some of the roles of the MHb in drug addiction are mediated by the connectivity with the LHb. However, the MHb to IPN connection is so prominent that other functions for the MHb that are not mediated by the LHb are likely important.

## Lesion studies

Given their small size and close proximity, electrically- or chemically-induced habenular lesions frequently affect both the MHb and LHb. Thus, it is often difficult to distinguish MHb and LHb function based on lesion data alone. In rodent studies, whole habenular lesions have been shown to cause cognitive impairment (spatial learning and memory), attention deficits, hyper-reactivity to stress, and schizophrenic-like symptoms (impaired performance in spatial memory) on the Morris Water Maze. These results prompted researchers to speculate about the possible involvement of the habenula in schizophrenia (Lecourtier et al., 2004). Lee and Goto (2011) demonstrated similar results after pharmacologically lesioning the MHb and LHb in rodents with ibotenic acid. Developmental disruption of prefrontal cortex volume, decreased dopamine (DA) receptor expression, and decreased DA transporter expression were observed and manifested through deficits in impulsivity, attention, and locomotion, respectively, suggesting the habenula's potential involvement in ADHD. These deficits were also seen when ibotenic acid was injected in the habenula and induced MHb-specific lesions, suggesting the MHb is responsible for the observed phenotypes rather than the “whole habenula” (Lee and Goto, 2011). When Gpr151 expressing neurons in the MHb were selectively eliminated using Cre-mediated expression of Eno2-STOP-DTA, there was a significant decrease of ACh in the IPN and a wide range of behavioral effects including: hyperactivity during the early night period, maladaptation to new environments, impulsive and compulsive behavior, delay and effort aversion in decision-making, deficits in spatial memory, reduced flexibility in complex learning paradigms, and lack of susceptibility to nicotine (Kobayashi et al., 2013). These findings suggest that neurons in the MHb are directly responsible for these behaviors and for regulating downstream expression of ACh. The development of tools and methods for selectively lesioning the MHb and MHb sub-fields opens the door to future avenues for studying the role of MHb in cognitive and behavioral processes.

## Mood and anxiety disorders

While the link between LHb hyperactivity and major depression disorder (MDD) receives the most scientific attention (Sartorius et al., 2010; Li Piriz et al., 2011), elevated activity in the MHb and IPN have also been linked to depression (Caldecott-Hazard et al., 1988). In rat studies of learned helplessness, a model of human MDD, researchers observed increased metabolic activity in the MHb, LHb, and IPN along with decreased metabolic activity in the VTA, basal ganglia, and amygdala (Shumake et al., 2003). These effects may be attributed to restricted brain expression of IL-18, a proinflammatory cytokine, in the superior subnucleus of the MHb (Sugama et al., 2002). Noradrenergic inputs into the MHb may trigger IL-18 (Aizawa et al., 2012). IL-18 is known to promote sleep in rabbits and rats (Kubota et al., 2001) and stimulates stress-induced immune responses that cause brain inflammation (Sugama et al., 2002). This stress response could lead to depression and other mood disorders (Dantzer et al., 2008). The inflammatory response caused by IL-18 may also cause degradation of the FR white matter pathway between the MHb and IPN (Felderhoff-Mueser et al., 2005). These white matter disruptions may play a role in schizophrenia (Sandyk, 1991; Ellison, 1994; Kelly, 1998).

The MHb may also play an important role in anxiety and fear (Yamaguchi et al., 2013). Severing the parallel pathways between the MHb and the TSN and bed nucleus of the anterior commissure in mice results in reduced anxiety and amplified fear responses (Yamaguchi et al., 2013). In zebrafish, nitroreductase lesions of the MHb increased fear-induced freezing behavior when electric shocks were paired with a red light compared to non-lesioned control fish (Agetsuma et al., 2010). Similarly, when light chain tetanus toxin (TeTXlc) expressing larval zebrafish were trained to swim away from a red light that predicted shock, fish with tetanus toxin-induced MHb lesions developed freezing behavior gradually while control fish did not develop freezing behavior (Lee et al., 2010). Mathuru and Jesuthasan buttressed these findings in a fear-inducing overshadow (simulation of a natural stressor—a predator) experiment. TeTXlc-expressing zebrafish with MHb lesions exhibited increased fear intensity as measured by increased time spent in the bottom quarter of the fish tank and increased episodes of pauses and slow swimming (Mathuru and Jesuthasan, 2013). Understanding the relationship between the MHb and both the LHb and IPN may reveal insight into depression and mood disorders and should be a future area of study.

## The re-birth of the medial habenula: nicotine studies

One of the most striking features of the MHb is the high level of the nAChR expression (McCormick and Prince, 1987; Quick et al., 1999). Historically, α4, α7, and β 2 subunit-containing nAChRs in the VTA, striatum, cortex, and hippocampus have been the focus of nicotinic research to the known role these structures play in nicotine addiction and to the high levels of nAChR expression in these areas (Picciotto et al., 1998; Franceschini et al., 2002; Maskos et al., 2005). The creation of nAChR gene knock-out (KO) mice has drawn attention to other nAChR subunits and to structures with high levels of other nAChR subunits, such as α3, b4 and α5 in the MHb or IPN (Cui et al., 2003; Salas et al., 2003, 2004a, 2009; Kedmi et al., 2004). Several lines of nAChR subunit KO mice were created and extensively characterized. The α3 and β 4 subunits were initially created to explore their importance in peripheral nervous system function. In fact, both α3 KO and β 2/β 4 double KO mice die within days of birth due to peripheral defects such as growth deficiency, megacystis, and mydriasis (Xu et al., 1999a,b). Historically, β 2 was considered the “addiction subunit,” since mice lacking this subunit did not self-administer nicotine (Picciotto et al., 1998; Picciotto and Corrigall, 2002). However, the α5 and β 4 subunits were shown to be necessary for nicotine-induced seizures and hypolocomotion, and somatic symptoms of nicotine withdrawal (Salas et al., 2004a,b, 2009). Heterozygous α3 mice also have shown decreased nicotine-induced seizures while β 2 KO mice showed normal somatic withdrawal, indicating subunit-specific related behaviors (Salas et al., 2004a). The α4 subunit was also identified as sufficient for reward, tolerance, and sensitization behaviors in mice (Tapper et al., 2004).

Symptoms of nicotine withdrawal can be induced in mice using chronic nicotine treatment followed by either sudden treatment cessation or mecamylamine (a broad spectrum nAChR antagonist) injection (De Biasi and Salas, 2008). Using this methodology, a role for the α2, α5, and β 4 subunits in the MHb and IPN during nicotine withdrawal was determined (Salas et al., 2004a, 2009). The α5 subunit is expressed in the MHb and high doses of nicotine are shown to stimulate the MHb-IPN tract and inhibit nicotine consumption. However, mice lacking the nAChR α5 subunit have exhibited decreased MHb to IPN input and lack nicotine-induced inhibition of the brain's reward system (Fowler et al., 2011). Similarly, elevated expression of the nAChR β 4 subunit increases nicotine aversion in mice by enhancing activity of the MHb to the IPN. However, nicotine aversion is reversed by increasing α5 expression in the MHb (Frahm et al., 2011). Thus we can conclude that a critical balance between α5 and β 4 expression may be important for habenular activity, and for nicotine consumption. Disruption of this balance in smokers may lead to high levels of nicotine use which may, in turn, selectively damage the FR.

Another avenue of MHb research utilizes pharmacological agents that specifically target the habenula. The African shrub Tabermanthe iboga contains ibogaine, an alkaloid with hallucinatory properties. ibogaine has a number of anti-addictive properties and has been shown to block rodent self-administration of cocaine (Cappendijk and Dzoljic, 1993), alcohol (Rezvani et al., 1995), and morphine (Glick et al., 1991). While ibogaine is banned in the United States and many other countries, a derivative of ibogaine (18-Methoxycoronaridine, 18MC) has been used to reduce the effects of nicotine, cocaine, alcohol, and amphetamine without major side effects (Glick et al., 2006; Taraschenko et al., 2007). 18MC is an antagonist that targets the highly-expressed β 4 subunit in the MHb and IPN (Glick et al., 2006). Taken together, these results suggest that nAChRs in the MHb are involved in multiple forms of addiction, including: nicotine, cocaine, methamphetamine, and alcohol. Pre-treatment with 18MC in rats may also block the observed increase of DA in the nucleus accumbens following acute nicotine treatment (Nisell et al., 1994; McCallum et al., 2012). Thus the MHb may also be functionally connected to DA release in the nucleus accumbens.

## Human studies

A few human neuroimaging studies have defined habenular activity (Ullsperger and von Cramon, 2003; Salas et al., 2010). However, the resolution of current MRI techniques does not seem to allow for the separation of signals from the MHb and LHb. Using 7T scanners (while most research is done in 3T scanners) it has been shown that the human medial and the LHb can be distinguished anatomically in *ex-vivo* brains, but not functionally *in vivo* (Strotmann et al., 2013a,b).

## Conclusion

This review addresses the importance of the MHb in regulating behavior and the involvement of this structure in multiple neuropathologies and addiction. A growing literature suggests that the MHb plays an important role in mood disorders, anxiety, stress, memory, and nicotine withdrawal as well as in cocaine, methamphetamine, and alcohol addiction. We strongly suggest that the MHb should no longer be neglected.

### Conflict of interest statement

The authors declare that the research was conducted in the absence of any commercial or financial relationships that could be construed as a potential conflict of interest.

## Abbreviations

MHb: Medial Habenula
LHb: Lateral Habenula
VTA: Ventral Tegmental Area
IPN: Interpeduncular Nucleus
FR: Fasciculus Retroflexus
Ach: Acetylcholine
Glu: Glutamate
nAChR: Nicotinic Acetylcholine Receptor
RMTg: Rostromedial Tegmental Nucleus
TSN: Triangular Septal Nucleus
DA: Dopamine
MDD: Major Depressive Disorder
TeTXlc: Light Chain Tetanus Toxin.
